# Isolation and Characterization of Two New Antimicrobial Acids from* Quercus incana (Bluejack Oak)*

**DOI:** 10.1155/2018/3798105

**Published:** 2018-01-28

**Authors:** Rizwana Sarwar, Umar Farooq, Sadia Naz, Nadia Riaz, Syed Majid Bukhari, Abdur Rauf, Yahia N. Mabkhot, Salim S. Al-Showiman

**Affiliations:** ^1^Department of Chemistry, COMSATS Institute of Information Technology, Abbotabad 22060, Pakistan; ^2^Department of Environmental Sciences, COMSATS Institute of Information Technology, Abbotabad 22060, Pakistan; ^3^Department of Chemistry, University of Swabi, Anbar, Khyber Pakhtunkhwa 23430, Pakistan; ^4^Department of Chemistry, College of Science, King Saud University, P.O. Box 2455, Riyadh 1451, Saudi Arabia

## Abstract

Two new compounds [1-2] were purified from ethyl acetate fraction of* Quercus incana.* The structure of these compounds is mainly established by using advanced spectroscopic technique such as UV, IR, one-dimensional (ID) and two-dimensional (2D) NMR techniques, and EI mass. The structural formula was deduced to be 4-hydroxydecanoic acid [1] and 4-hydroxy-3-(hydroxymethyl) pentanoic acid [2]. Both isolated compounds were tested for their antimicrobial potential and showed promising antifungal activity against* Aspergillus niger* and* Aspergillus flavus*.

## 1. Introduction

The family Fagaceae is large family comprising 8 genera and about 800–1100 species.* Quercus* is the largest genus of family Fagaceae having huge medicinal importance and mostly found in dry conditions [1]. The genus* Quercus* have long been considered among the clades of woody angiosperms in terms of species diversity, horticultural merit, ecological dominance, and industrial and economic values [2]. The* Quercus robur *is the only cultivated species while other 600 known species are found in temperate regions of the Northern Hemisphere, Southward through Central America to Colombia and through Turkey to Pakistan [3].

The wood is durable, is attractively grained, and is mostly utilized for timber purposes; it is particularly important in shipbuilding, construction for flooring, furniture, railroad ties, and veneers. The bark of* Quercus* spp. has been used for medicinal purposes and is an important source of phenolic compounds like tannins which are used for tanning leather and wine production [4]. The fruit (acorn) of* Quercus* has husk coating which is edible and highly nutritious and rich in carbohydrates and protein.* Quercus* (oak) species are utilized in conventional pharmaceutical, as astringent, antiseptic, and hemostatic and in addition to the treatment of acute diarrhea, hemorrhoid, and oral, genital, and anal mucosa inflammation. Moreover, the decoction plants from this genus can be used against burns and added to ointments for the healing of cuts [5]. Oak seeds are a major source of sugar, amino acids, lipids, and different sterols [6].* Quercus *species have been utilized against problems of skin, wounds and gastrointestinal illnesses [7], astringent, mellow germ-free, small cuts [8], and mouth washes [9] all suggesting their antimicrobial potential.

Genus* Quercus* is characterized by six species found mostly in Northern areas of Pakistan. The most promising timber specie is* Quercus incana* Roxb. (Blue jack oak or cinnamon oak) locally called Ban shindar, Kharpata serci (Punjabi), Rein (Hindko), and Serie (Pushto) [10]. The* Q. incana* has huge medicinal usage; it may be used as astringent [11], diuretic, and antidiarrheal agent and for treatment of asthma. Bark and leaves of* Q. incana* may be used as antipyretic, antirheumatism, antidiabetic, and antiarthritic purposes [12]. These medical applications and therapeutic potential of* Quercus incana* prompted us to carry out the phytochemical investigation to explore biologically active compounds.

## 2. Material and Methods

### 2.1. Experimental Procedures

The ethyl acetate soluble fraction was selected for isolation of bioactive compounds using column chromatographic analysis having column silica and flash silica gel as an adsorbent material. The column was eluted by using* n*-hexane and ethyl acetate with increasing polarity, which yield two new compounds 4-hydroxy decanoic acid [**1**] and 4-hydroxy-3-(hydroxymethyl) pentanoic acid [**2**] by increasing polarity. The purity of compounds [**1-2**] was checked by using precoated TLC plates. The IR spectrum was recorded by using spectrophotometer JASCO-320A. The EI mass was recoded on double focusing Varian MAT-312 Spectrometer. ^1^H-NMR and ^13^C-NMR spectra were measured by using advance Bruker AMX-300 spectrometer machine. The chemical shifts in parts per million (*δ*) relative to tetramethylsilane as an internal standard and scalar* (J)* were described in Hz.

### 2.2. Plant Extraction and Fractionation

Extraction and fractionation of* Quercus incana* were reported in our previous study [13]. Ethyl acetate soluble fraction was subjected to repeated column chromatography which yielded two pure compounds [**1**-**2**].

### 2.3. Antibacterial Activity of Compounds [1-2]

Antibacterial activity was performed by agar well diffusion method with some modifications [14]. Three Gram-positive (*Staphylococcus aureus, Micrococcus luteus, *and* Bacillus subtilis*) and Gram-negative (*Escherichia coli, Pseudomonas pickettii,* and* Shigella flexneri*) pathogens were used in study. 10 *µ*g of each compounds [**1**-**2**] was dissolved in 1 mL DMSO. Standard drug and each sample (20 *μ*L) were poured in 6 mm well. The assay plates were incubated at 37°C for 24 hrs. The zone of inhibition was dignified in mm and DMSO was used as a negative control in the experiment.

### 2.4. Antifungal Assay

Disc diffusion methods were used for determination of antifungal effects by using two selected fungal strains such as* Aspergillus niger *and* Aspergillus flavus* [15]. DMSO was used as a solvent; before applying compounds on petri plates DMSO was completely evaporated.

*Characterization of Compound 1*. Colorless oil; IR (KBr) *υ*_max_ 3622 br (OH), 1714 (C=O) cm^−1^. [*α*]_D_^25^ + 34.80° (*c* = 0.78, CHCl_3_). EI-MS *m*/*z*: (rel. int.) 188 [M]^+^ (15), 176 (9), 157 (35), 128 (5), 115 (9). HR-EI-MS: *m*/*z* 188. 1420 (calcd. for 188.1412 for C_10_H_20_O_3_). ^1^H-NMR (CDCl_3_, 300 MHz): *δ* 3.62 (1H, m, H-4), 0.88 (3H, t,* J* = 7.7 Hz, H-10), 2.08 (2H, m, H-2), 1.49, 1.62 (2H, m, H-3), 1.45 (2H, m, H-5), 1.47, 1.33 (2H, m, H-6), 1.30 (2H, m, H-7), 1.28 (2H, m, H-8), 1.29 (2H, m, H-9). ^13^C-NMR (CDCl_3_, 75 MHz): *δ* 179.6 (C-1), 72.1 (C-4), 13.9 (C-10), 34.6 (C-2), 34.9 (C-3), 37.9 (C-5), 26.1 (C-6), 29.8 (C-7), 32.0 (C-8), 22.1 (C-9).

*Characterization of Compound 2*. Colorless oil: IR (KBr) *υ*_max_ 3495 (OH), 1708 (C=O) cm^−1^. [*α*]_D_^25^ + 53.60° (*c* = 0.97, CHCl_3_). EI-MS *m*/*z*: (rel. int.%) 131 [M-OH]^+^ 131 (10), 115 (8), 86 (100), 71 (31). HR-EI-MS: *m*/*z*  [M-OH]^+^ 131.0744 (calcd. 131.0736 for C_6_H_11_O_3_-OH). ^1^H-NMR (CDCl_3_, 300 MHz): *δ* 2.35 (1H, m, H-2), 2.04 (1H, m, H-2), 2.58 (1H, m, H-3), 3.92 (1H, m, H-4), 1.23 (3H, d,* J* = 6.9 Hz, H-5), 4.31 (2H, m, H-1′).^13^C-NMR (CDCl_3_, 75 MHz): *δ* 177.5 (C-1), 27.3 (C-2), 45.9 (C-3), 68.1 (C-4), 20.9 (C-5), 65.5 (C-1′).

## 3. Result and Discussion

**Figure 1:**
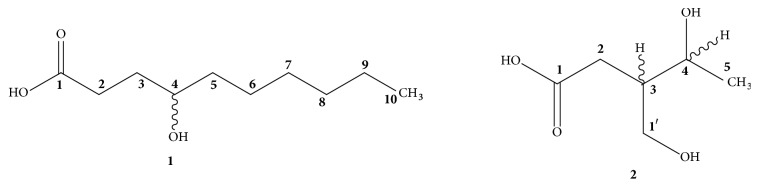
Structure of compounds [**1-2**].

Ethyl acetate soluble fraction was subjected to repeated column chromatography on silica gel using* n*-hexane and ethyl acetate as a solvent with gradual increasing in polarity up to 100% ethyl acetate, which resulted in four subfractions (Fractions** A**–**D**). The fractions obtained based on TLC profile were resubjected to pencil column chromatography and eluted with* n*-hexane : EtOAc, 25 : 75 and* n*-hexane: EtOAc, 30 : 70 to purify compound** 1 **(10.5 mg) and compound** 2** (9.8 mg) (Figure 1).

Compound** 1** was isolated as a colorless oil and has molecular formula of C_10_H_20_O_3_ as suggested by molecular ion peak at *m*/*z* 188 [M]^+^ in HR-EIMS. The other fragment peaks were obtained at *m*/*z* 176, 157, 128, and 115. The HR-EIMS gave exact mass of compound** 1** which was at *m*/*z* 188.1420 (calcd. *m*/*z* 188.1412). The IR spectrum displayed absorption bands for hydroxyl and carbonyl groups at 3622 and 1714 cm^−1^, respectively. The ^1^H-NMR spectrum of compound** 1 **exhibited typical signal for aliphatic acid skeleton, which was strongly supported by DEPT experiment. The ^13^C-NMR spectrum revealed the presence of one methyl, one methine, seven methylene, and one quaternary carbon signals. The methine signal appearing at *δ*_H_ 3.62 (1H, m) was assigned to H-4 while methyl group resonated at *δ*_H_ 0.88 (3H, t,* J* = 7.7 Hz). Typical methylene signals were resonated as multiplet for seven methylene carbons at *δ*_H_ 2.08 (2H, m), *δ*_H_ 1.49 (1H, m), *δ*_H_ 1.45 (2H, m), *δ*_H_ 1.33 (1H, m), *δ*_H_ 1.47 (1H, m), *δ*_H_ 1.30 (2H, m), *δ*_H_ 1.28 (2H, m), and *δ*_H_ 1.29 (2H, m) assigned to H-2, H-3, H-5, H-6, H-7, H-8, and H-9, respectively (Table 1). The ^13^C-NMR spectrum (BB and DEPT) corroborated the presence of seven methylene carbons, one methine carbon, one terminal methyl carbon, and one quaternary carbon. The carbonyl carbon showed signals at *δ*_C_ 179.6 whereas methine signal centered at *δ*_C_ 72.1. The ^13^C-NMR chemical shift of CH_3_-C10 was observed at *δ*_C_ 13.9 and seven methylene carbons appeared at *δ*_C_ 34.6, 34.9, 37.9, 26.1, 29.8, 32.0, and 22.1 for C-2, C-3, C-5, C-6, C-7, C-8, and C-9, respectively (Table 2). The HMBC and COSY spectra were quite helpful for accurate placement of various substituents in the molecule. The HMBC spectrum showed strong correlation of methine proton at *δ*_H_ 3.62 (H-4) with C-2, C-3, C-5, and C-6 [13]. The methyl proton at *δ*_H_ 0.88 showed strong HMBC correlation with C-9 (*δ*_C_ 22.1), C-8 (*δ*_C_ 32.0) which was quite supportive in the establishment of structure. Finally all spectral data confirmed, compound** 1** as an aliphatic acid having straight chain of -(CH_2_)_7_-CH_3_- moiety [16] and was proposed to be 4-hydroxy decanoic acid.

Compound** 2 **was isolated as colorless oil. Its structure was mainly established by ^1^H-NMR and high resolution mass spectroscopy and supported by ^13^C-NMR spectrum. Its molecular formula C_6_H_12_O_4_ was concluded from the accurate mass measurement of peak at *m*/*z*  [M-OH]^+^ 131, corresponding to molecular composition C_6_H_11_O_3_-OH. In addition to its molecular ion peak, it showed some characteristic fragments at *m*/*z* 115, 86, and 71. The HR-EIMS gave exact mass of compound** 2** at *m*/*z* 131.0744 (calcd. 131.0736 for C_6_H_11_O_3_-OH). The IR spectrum showed absorption bands at 3495 cm^−1^ and 1708 cm^−1^ indicating presence of the hydroxyl group and carbonyl carbon, respectively. Similarly, broad absorption centered at 2935 cm^−1^ suggested the presence of carboxylic acid. The ^1^H-NMR showed a signal for a secondary methyl group at *δ* 1.23 (3H, d,* J* = 6.9 Hz, H-5), connected to a methine group resonated at *δ*_H_ 3.92 (1H, m, H-4) possessing hydroxyl group, while the other methine signal appeared at *δ*_H_ 2.58 (1H, m, H-3). The downfield methylene bearing hydroxyl group appeared at *δ*_H_ 4.31 (2H, m, H-1′) and the other methylene centered at *δ*_H_ 2.04 (1H, m, H-2) and *δ*_H_ 2.35 (1H, m, H-2) was directly connected to carboxylic acid (Table 1). The ^13^C-NMR spectrum confirmed the presence of one methyl carbon, two methylene groups, two methine carbons, and one quaternary carbon in the structure.

**Figure 2:**
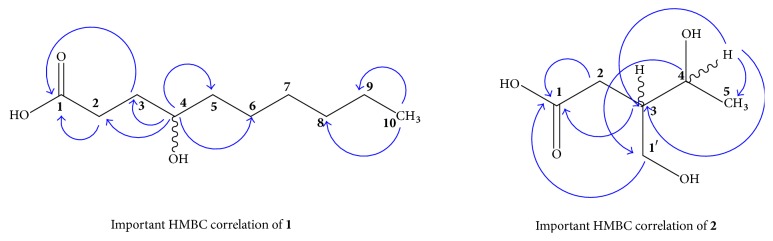
HMBC correlation of compounds [**1-2**].

In ^13^C-NMR spectrum, signal for secondary methyl appeared at *δ*_C_ 20.9 whereas the methine signal bearing hydroxyl group was observed at *δ*_C_ 68.1 for C-4. The signal for another methine appeared at *δ*_C_ 45.9 for C-3. The side chain methylene having free hydroxyl group resonated at *δ*_C_ 65.5, while the second methylene group at position C-2 appeared at *δ*_C_ 27.3. Similarly, the quaternary carbon in the form of carboxylic acid showed signal at *δ*_C_ 177.5 (Table 2). Based on the HMBC and H-H COSY correlation (Figure 2), the connectivity of the C-1 to C-5 chain was found in agreement with literature [17]. The HMBC spectrum showed H-C correlation of CH_3_-5 with that of C-4 and C-3. Similarly the position of hydroxyl group at C-1′ was confirmed by strong HMBC correlation of CH_2_-1′ with C-3, C-2, and C-4 and weak interaction with C-1. The structure of compound** 2** was mainly established by ^1^H-NMR, high resolution mass spectrometry and supported by ^13^C-NMR spectrum. From all spectral data it was evident that compound** 2 **was 4-hydroxy-3-(hydroxymethyl) pentanoic acid.

### 3.1. Antibacterial Activity

The antibacterial activity of isolated compounds [**1-2**] was determined by agar well diffusion method (Table 3). Compound** 1** was significantly active against* Bacillus subtilis, Staphylococcus aureus,* and* Micrococcus luteus* (Gram-positive). Both compounds [**1-2**] showed promising antibacterial activity against* Staphylococcus aureus *with 16 mm and 13 mm zone of inhibition. Compound** 2** was moderately active against* Bacillus subtilis *and* Micrococcus luteus *with 5 mm and 9 mm zone of inhibition. Both compounds were inactive against* Escherichia coli *and* Shigella flexneri*.

### 3.2. Antifungal Activity

Antifungal activity of both compounds [**1-2**] was done against* Aspergillus flavus* and* Aspergillus niger*. Both compounds [**1-2**] showed immense activity against* Aspergillus niger* with 15 mm ± 0.70 and 22 mm ± 0.57 zone of inhibition (Table 4). Moderate activity was observed by compound** 1** against* Aspergillus flavus *having 12 mm ± 0.50 zone of inhibition.

## 4. Conclusion

The current study describes the isolation, characterization, and antimicrobial activity of isolated compounds from ethyl acetate fraction of* Quercus incana*. Both compounds displayed promising antimicrobial activity against human bacterial and fungal strains. Therefore, these isolated compounds may be considered as the lead compounds as an antimicrobial agents.

## Figures and Tables

**Table 1:** ^1^H-NMR (CDCl_3_, 300 MHz) data of compounds [**1-2**] in ppm, *J* in Hz.

Position	**1**	**2**
1	-	-
2	2.08, m	2.04, m 2.35, m
3	1.62, m 1.49, m	2.58, m
4	3.62, m	3.92, m
5	1.45, m	1.23 (1H, d, *J* = 6.9 Hz)
6	1.47, m 1.33, m	-
7	1.30, m	-
8	1.28, m	-
9	1.29, m	-
10	0.88 (t, *J *= 7.7)	-
1′	-	4.31, m

**Table 2:** ^13^C-NMR (CDCl_3_, 75 MHz) of compounds [**1-2**] in ppm.

Position	**1**	**2**
1	179.6	177.5
2	34.6	27.3
3	34.9	45.9
4	72.1	68.1
5	37.9	20.9
6	26.1	-
7	29.8	-
8	32.0	-
9	22.1	-
10	13.9	-
1′	-	65.5

**Table 3:** Antibacterial activity of isolated compounds [**1**-**2**].

S. number	Culture	Zone of inhibition (mm)		
		**1**	**2**	Ciprofloxacin
1	*Bacillus subtilis*	8	5	8
2	*Staphylococcus aureus*	16	13	16
3	*Micrococcus luteus*	11	9	18
4	*Pseudomonas pickettii*	0	0	0
5	*Escherichia coli*	0	0	0
6	*Shigella flexneri*	6	9	14

**Table 4:** Antifungal activity of isolated compounds [**1**-**2**].

Extract	Pathogenic fungi	
	*Aspergillus flavus *	*Aspergillus niger*
1	12 mm ± 0.50	15 mm ± 0.70
2	17 mm ± 0.28	22 mm ± 0.57
Nystatin (standard)	16 mm ± 0.92	21 mm ± 0.28

*Note*. Each value in the table was obtained by calculating the average of three experiments.
